# "The Hospital" Medical Book Supplement No. XXXIII

**Published:** 1910-09-17

**Authors:** 


### "THE HOSPITAL"

### Medical Book Supplement No. XXXIII.

CONTENTS.

| | Page |
|---|---|
| **Medicine—** | |
| Spa Treatment and the Choice of a Suitable Spa ... ... | 739 |
| Hypnotism and Suggestion in Daily Life and Medical Practice | 739 |
| **Gynæcology—** | |
| The Principles of Gynæcology... ... ... ... ... ... | 740 |
| **Public Health—** | |
| Gorton Urban District Council ... ... ... ... ... | 740 |
| The Annual Report on the Health, Sanitary Condition, etc., etc., of the County Borough of Hastings for the Year 1909... | 740 |
| **Pharmacology—** | |
| Synthesis of Hordenine, the Alkaloid from Barley ... ... | 742 |
| The Active Principle of a Bini Spear Poison ... ... ... | 742 |
| Isolation and Synthesis of *p*-Hydroxyphenylethylamine, an Active Principle of Ergot Soluble in Water ... ... ... | 742 |
| Further Synthesis of *p*-Hydroxyphenylethylamine ... ... | 742 |
| The Alkaloids of Ergot—Part II. ... ... ... ... ... | 742 |
| The Cultivation and Biological Characters of Bacillus Acnes... | 742 |
| Organic Analysis Chart ... ... ... ... ... ... ... | 742 |
| **School Hygiene—** | |
| Annual Report to the Education Authority on School Hygiene for the Year 1909 ... ... ... ... ... ... ... | 74[illegible] |
| Medical Examination of Schools and Scholars ... ... ... | 74[illegible] |

### MEDICINE.

Spa Treatment and the Choice of a Suitable Spa. By Neville Wood, M.D., M.R.C.P. (London: Adlard and Son. 1910. Pp. 59. Price 2s. net.)

The author is strictly impartial in dealing with both English and foreign spas, which, as he points out, should not be in competition with, but complementary to, each other. Foreign spas are suitable to persons of sedentary, and English to those of active habits. He shows the merits of each resort and does not hesitate to point out any defect it may possess. We can heartily recommend this interesting and well-written monograph, not only to those who are concerned in the subject with which it deals, but to practitioners in general, who will find much useful information within its few pages.

Hypnotism and Suggestion in Daily Life and Medical Practice. By Bernard Hollander, M.D. (London: Pitman and Sons, 1 Amen Corner, E.C. 6s. net.)

Dr. Hollander is well qualified to write on the subject he has chosen, for he may be regarded as an authority upon hypnotism in this country. The subject, be it added, is one that medical men, and especially general practitioners, are too apt to ignore. For hypnotism has still, to many people, a suspicion of charlatanism around it, although it is true that we are gradually coming to look upon it as a legitimate means of cure and alleviation in certain conditions. On the Continent and America it has long since come into its own as a recognised method which may be justifiably employed in suitable cases. The question to be determined still is, What are those conditions, and how is the method to be used? To those that want a simple, straightforward answer to these questions we can confidently recommend Dr. Hollander's handbook. He deals with the subject in a lucid manner, discussing it as fully as the limitations of his space allow. Perhaps he takes too much for granted as regards the understanding of the average reader. The science of mind is, unfortunately, not taught in our medical schools, and the average practitioner, who has not made a study of psychology, may find himself at sea and out of his depth before he has waded through the first chapter on "The Subconscious Mind." But that, after all, is not the author's fault. A writer who deals with such a subject as this must necessarily take it for granted that his readers possess a certain general acquaintance with the principles of psychology, and, honestly speaking, we cannot assert that Dr. Hollander is anywhere abstruse in this book. As a popular exposition of hypnotism it is perhaps somewhat too deep for the average reader; as a scientific exposition, meant for the elect, it is by no means full enough. But for the intermediate section, for those, that is, who already know something of the matter and desire fuller explanations, it is admirably suited.

The first few chapters deal with general subjects, with the history and phenomena of hypnotism, clairvoyance, telepathy, and allied matters. They are conscientiously and clearly written, and show that the author has closely studied and worked up his material. We cannot say that they contain anything that is novel or original, but we do not think that Dr. Hollander will claim that his work is in any sense pioneering. The chapter on English hypnotists is full and accurate, and, indeed, an excellent summary of the work done in this country up to the time of the British Medical Association's Special Investigation Committee in 1890. That on the French schools is even fuller, and contains much interesting information. One special point, for example, is entirely new to us, and may be new to most of our readers. We give it in Dr. Hollander's own terms: "In Austria the law requires army horses to be mesmerised for the purpose of shoeing them. This was introduced by a cavalry officer named Balassa, and hence it has been termed and is known as the 'balassiren' of horses. By this method horse-tamers tame the wildest colts and the most vicious horses in an hour." It would be highly interesting to know the experience of veterinary surgeons in this country in the matter. The hypothesis that hibernation is an auto-hypnosis is alluded to, and the general conditions of mesmerism are fully discussed. In the concluding chapters Dr. Hollander deals with the cure of mental and nervous disorders by suggestion and hypnotism. His experience has been wide and his illustrative cases cited are therefore highly interesting and instructive. He has found hypnotism of prime value in hystero-epileptic and neurasthenic conditions, and especially in cases of drug habit or mental aberration. The chapter on mental healers is particularly worth consideration on account of the succinct historical synopsis given. We are glad to note that the author lays stress on the importance of combining hygienic treatment with hypnotism, for there is a tendency to look upon suggestion as a sufficiently powerful remedy by itself, which is far from being the case. The book has a good index and is well printed and bound. It is eminently readable, and is well worthy of a place on every practitioner's bookshelf, for it is really one of the best works on the subject that we have read in English, and there is no doubt that it supplies a decided want.

### GYNÆCOLOGY.

The Principles of Gynæcology. By W. Blair Bell, M.D. (London: Longmans, Green and Co. 1910. Pp. 551. Price 21s. net.)

There have been a great many text-books on this subject published within recent years; partly, no doubt, because of the constant progress in the science of gynæcology, and partly also because no one book has commanded such extensive approbation as to outdistance all competitors. Dr. Blair Bell here presents a work designed for practitioners and students, and it is quite clear that his book will obtain very considerable popularity with both classes; more especially, perhaps, with the former, on account of the thoroughly practical handling of the subject and the way in which the difficulties of treating and diagnosing patients in private practice are appreciated and dissipated. The book is written in a most readable way; the style is clear and lucid, the illustrations are in general excellent, print and paper are of the best; there is a splendid index; in fact, no pains have been spared to set off to the best advantage the actual text, itself always instructive and admirably arranged. Turning to some of the separate sections, it is first to be noted that the large subjects of fibroids, cysts, and growths generally are allotted space commensurate to their importance. If any criticism can here be made, it is that not quite enough stress is laid on the early diagnosis, symptoms, and signs of malignant diseases of the uterus; but in the main these chapters are excellent. In the sections on the examination of the patient are reproduced some of Head's figures showing hyperæsthetic areas connected with the ovary and Fallopian tube; the somewhat meagre reference to these in the text fails to note the very important point that areas of hyperæsthesia such as those depicted are much more often the indication of trouble in the appendix than in the reproductive organs; the figures would be better omitted altogether if there is not sufficient space for entering more fully into this somewhat complicated question. The difficult problems of uterine displacements are treated with fair success. This is always a part of gynæcology which puzzles students, because many of the current text-books are so vague about it; and there are few practitioners who are averse to picking up any fresh wrinkles about these troublesome complaints. Dr. Bell is more successful than most authors; but even he loses something of his customary perspicuity in dealing with this topic. He describes and figures an ingenious douche which seems to present marked superiority over the patterns in common use in London. For menstrual derangements the author's great standby is calcium; this he recommends for menorrhagia and for amenorrhœa, as well as for dysmenorrhœa. Recognising the apparent inconsistency of this, he offers a theoretical explanation which does not carry much conviction. If calcium lactate will really cure with certainty the various disorders for which it is here recommended, so much the better for patient and doctor; but some alternative treatment to be tried should this drug fail might well have been suggested. In ectopic gestations the difficulty of deciding whether to operate at once on a patient almost pulseless or to wait for a rally is recognised; on the whole Dr. Bell is in favour of immediate operation, as against leaving the patient to get over the period of collapse. On operative gynæcology there are some excellent chapters. Due attention to preparation and to after-treatment are insisted upon, and a strong plea for open ether as an anæsthetic is registered. The consideration of operative procedures is confined to a brief *résumé* of the essential principles and details of the chief methods employed in uncomplicated cases. Then follows an appendix by Dr. Curtis Webb on electro-therapeutics in gynæcology; this is tantalisingly short, and deals almost exclusively with the kinds of electricity used. A little more space might well have been afforded in which this acknowledged authority could have dealt with the indications for electrical treatment, and the results of it in various diseases. A second appendix is a classification of the causes of certain symptoms drawn up after the fashion of the well-known "Index of Symptoms" by Dr. Leftwich. The classification is very complete and well done, and is a departure from routine which is calculated to be extremely useful on occasions in general practice. Dr. Bell is certainly to be thanked for a contribution to gynæcological literature which is original and full of vitality; it may be confidently expected that his book will speedily establish a reputation as one of the soundest and most practical manuals on this subject yet published.

### PUBLIC HEALTH.

Gorton Urban District Council: Annual Report of the Medical Officer of Health and Sanitary Inspector for the year 1909. (Manchester: Henry Blacklock and Co., Ltd. 1910. Pp. 22.)

The Urban District of Gorton ceased to exist as such on November 8, 1909, after which date amalgamation with Manchester took place. Statistics and figures are, however, also given for the whole year by arrangement. The population of the district was estimated at 41,000 at the middle of the year. The number of births was 1,038 (males 525, females 513) up to November 8, and 1,153 for the whole year. The birth-rates worked out respectively at 29.5 and 28.1 per thousand, as compared with 33.3 for the previous five years. Eighteen births were registered as illegitimate. The general, zymotic, and infantile death-rates worked out respectively at 14.9, 1.6, and 139 per thousand for the year, as compared with an average of 17.4, 2.6, and 164 for the previous five years. There were 32 notifications to the medical officer of health of phthisis occurring in paupers during the year. Death from consumption and other tuberculous diseases formed 11.9 per cent. of the total deaths of the district. Other causes of death during the year were measles 34, whooping cough 3, scarlet fever 4, diphtheria 13, diarrhœa 11, typhoid fever 2, puerperal fever 5, phthisis 44, tuberculous disease 29, respiratory diseases 117, cancer 31, premature birth 19, alcoholism and cirrhosis of the liver 5, heart disease 40, accidents 11, influenza 4, suicide 4, convulsions 16, wasting diseases 28, old age 35. The report of the sanitary inspector, which brings the pamphlet to a close, does not call for special mention.

The Annual Report on the Health, Sanitary Condition, etc., etc., of the County Borough of Hastings for the Year 1909. By A. Scarlyn Wilson, M.A., M.B., D.P.H.Cantab., M.R.C.S.Eng., Medical Officer of Health. (Eley, Starkey and Co., Ltd., St. Leonards-on-Sea. 1910.)

The County Borough of Hastings has an area of 4,857 acres, including 373 acres of foreshore. The population at the last census was 65,528, and in the year under review the birth-rate was 15.24, as compared with a death-rate of 12.39 per thousand, both rates being below the average for the past decade, which were respectively 18.01 and 13.54 per thousand. Of the births 6.5 per cent. were illegitimate, which is below the average. The rate of infantile mortality was only 76 per thousand of registered births, which is much below that of the large towns. Owing to the fact that the chief industry of the district is the letting of lodgings, there is a large preponderance of female over male inhabitants at all age periods over fifteen years. It is from this lack of male employment that difficulties arise in the housing of the working classes, and not from scarcity of suitable accommodation; which, on the other hand, is good as well as ample. The rainfall for the year was 31.43 inches, which is more than two inches above the average, in contrast with 1,876 hours of sunshine, or ninety-three above the annual average. Owing to the faults and folds in the geological strata much expense is incurred in the provision of adequate water supplies, which are mainly obtained from wells and headings in the Ashdown Sands. When obtained the water is of excellent quality, being soft and bacteriologically extremely pure.

### SCHOOL HYGIENE.

ANNUAL REPORT TO THE EDUCATION AUTHORITY ON SCHOOL HYGIENE FOR THE YEAR 1909. By J. COOTE-HIBBERT, M.D. (Lond.), D.P.H., Medical Officer of Health and School Medical Officer to the County Borough of Warrington. (Warrington: Mackie and Co., Ltd. 1910.)

DURING the year 4,606 children were medically examined. Of these 11.3 per cent. of boys and 34.1 of girls were found to have pediculi capitis. Defective vision was found in 1.92 of boys and 3.73 per cent. of girls, the apparent lowness of the percentage in each case being due to the fact that only children who could not see 6/18 Snellen's types were returned as defective. Defective teeth were noticed in 5.7 per cent. of boys and 4.71 of girls, the standard of defectiveness being taken as the possession of four or more carious teeth. Ear disease was found in 1.5 per cent. of both sexes. The report contains a series of elaborate tables in which each school is dealt with separately as regards its site, playground, sanitary conveniences, lighting, ventilation, heating, etc.

MEDICAL EXAMINATION OF SCHOOLS AND SCHOLARS. Edited by T. N. KELYNACK, M.D. (P. S. King and Sons, Orchard House, Westminster. 10s. 6d. net.)

A LARGE literature has accumulated during the last few years upon the subjects with which this volume deals, and it has grown rather unwieldy and chaotic, so that there seems every reason for a book which should serve as a guide to the practitioner who wishes to know where to find special articles and to have at hand a convenient manual for reference purposes. This object is fulfilled by Dr. Kelynack's book, which, although it is not so full as one might wish, and although it gives evidence of hurried compilation, is nevertheless a useful practical handbook. Like every work that Dr. Kelynack has been associated with as editor, it is admirably edited, and the bibliographies appended to each section and the very full index are excellent.

The book contains thirty-two essays in all, and these are of widely different interest and merit, and are prefaced by a well-written introduction by Sir Lauder Brunton, which, however, contains nothing very original or striking. Obviously the most important chapters are those likely to prove helpful to school doctors. One of these is Dr. Riviere's excellent article on the Routine Examination of School Children. We are glad to find that Dr. Riviere lays stress on the non-desirability—to put it mildly—of finger examinations of the naso-pharynx. Every hospital surgeon knows that a large number of school children are sent to the out-patient department for treatment of comparatively trivial naso-pharyngeal lesions, and it is high time that attention should be drawn to the fact that adenoids and tonsils are not likely to "cause serious trouble to the child in after life" unless they give rise to obstruction or are already responsible for such complications as would entail their removal. And in this connection it may also be pointed out that where any operation on such children is necessary it should be done thoroughly. It is the height of folly to remove a slice of each tonsil and to take away the central pad of adenoids, leaving the lateral fossæ filled up, and it is even more foolish to think that removal without subsequent education in breathing exercises, swimming, etc., is likely permanently to benefit the child. We wish Dr. Riviere had been more didactic in regard to thoracic lesions. His section dealing with the heart is, on the other hand, all that can be desired—clear, concise, and to the point. Only it is questionable if the average school doctor has the skill and the patience to map out the cardiac dulness systematically in the five minutes that are given him for testing the physical fitness of the scholar. Most doctors will content themselves with auscultatory evidence, which, for all practical purposes, is sufficient to demonstrate gross lesions if present. The chapters on the medical inspection of Army schools and scholars by Lieut.-Col. Melville, of children under the Poor Law by Dr. Edwards, of girls in secondary schools by Dr. Corthorn, and of mentally defective children by Dr. Tredgold are all good. Dr. Duke's able chapter on the examination of public school boys is one of the best in the book, and the writer's vast experience and broadminded outlook make his article of particular value.

There is necessarily a certain amount of overlapping in the various subjects, but on the whole that is much less than one expected, for Dr. Kelynack has done his work as editor very carefully. The chapters dealing with medical inspection in Germany, France, Norway and Sweden, Denmark, Switzerland, Ireland, Canada, New Zealand, and Australia are all instructive, and give much valuable comparative information. The article on "The Feeding of the School Child" by Dr. Lambert is a clear exposition of the whole question, and will repay careful perusal. That on "School Clinics" by Dr. Lewis Williams deals with a debatable subject, and it is possible that some of us may not be willing to follow the writer in his conclusions. To our mind, however, it is one of the clearest and best reasoned pleas for the establishment of such centres for treatment that we have seen, and those who are opposed to these innovations would do well to read it attentively. The subject is one of great importance, and we hope to deal with it elsewhere on a future occasion. We much regret that want of space forbids us reviewing Dr. Kelynack's manual with the fulness it deserves. Sufficient has been said, however, to indicate its usefulness and practical value to the practitioner interested in the medical inspection of school children. It is a unique volume, and should prove of service to a great number of men and women who have had added responsibilities thrust upon them in consequence of the State having at last realised its obligations in regard to the physical inspection of its growing citizens.

### PHARMACOLOGY.

Synthesis of Hordenine, the Alkaloid from Barley. By George Barger, M.A., D.Sc. (From the Wellcome Physiological Research Laboratories, Herne Hill, London, S.E.)

This crystalline alkaloid was isolated from barley germs by Léger. Dr. Barger has recently synthesised *p*-hydroxyphenylethylamine, a substance which is closely related chemically to hordenine. The present paper deals with his experiments to synthesise the latter from the former.

The Active Principle of a Bini Spear Poison. By P. P. Laidlaw, M.A., B.C. (From the Wellcome Physiological Research Laboratories, Herne Hill, London, S.E. 1909.)

The author has had the opportunity of examining two spear-heads poisoned for elephant hunting obtained from West Africa. The huntsman from whom they were obtained would not disclose the actual ingredients used in the manufacture of the poison, which is kept secret and handed down from father to son, but stated that they were made from vegetable matter, took three months to prepare, and retained their virulence for years. The author made a series of experiments with various extracts of the brownish green gluey mass with which the spears were covered, and came to the conclusion that the active principle of the poison belongs to the class of glucosides, gives the characteristic colour-reaction of Kombé strophanthin, and possesses the solubilities and physiological properties of that substance. The fact that he was unable to obtain crystalline strophanthidin from the poison he attributes to the presence of impurities.

Isolation and Synthesis of *p*-Hydroxyphenylethylamine, an Active Principle of Ergot soluble in Water. By George Barger, M.A., D.Sc.

Further Syntheses of *p*-Hydroxyphenylethylamine. By George Barger, M.A., D.Sc., and George Walpole, B.Sc., A.I.C. (Both from the Wellcome Physiological Research Laboratories, Herne Hill, London, S.E.)

The author of the first monograph in association with Walpole has recently isolated *p*-hydroxyphenylethylamine from putrid meat, and its physiological properties were such as to suggest that this base might be the active principle of aqueous ergot extracts. He has been able to show that the base occurs in such extracts, and that its presence accounts satisfactorily for such activity as is not due to the presence of ergotoxine. In the second monograph the same author, in association with G. S. Walpole, describes two further methods by which the base can be synthetically prepared. In the first phenylethylamine was used, and in the second anisaldehyde as starting-points.

The Alkaloids of Ergot. Part II. By George Barger, M.A., D.Sc., and Arthur James Ewins, B.Sc. (From the Wellcome Physiological Research Laboratories, Herne Hill, London, S.E.)

In a former communication one of the authors, in conjunction with F. H. Carr, described the new amorphous alkaloid ergotoxine, $C_{35}H_{41}O_6N_5$, and assigned the formula $C_{35}H_{39}O_5N_5$ to Tanret's crystalline ergotinine. The crystalline alkaloid was thus proved to be the anhydride of the amorphous one as was first surmised by Kraft. Boiling with methyl alcohol will transform the former into the latter, but when the latter is warmed with very dilute phosphoric acid a crystalline phosphate of an amorphous base is formed which, while closely resembling ergotoxine sulphate in physiological action and having the same melting-point, is entirely different to it. The cause of the difference has now been found by the authors of the present monograph. When ergotinine is heated with phosphoric acid in ethyl alcohol, the phosphate of ergotoxine ethyl ester is formed, which crystallises in plates and not in thin needles as does ergotoxine sulphate. Thus ergotoxine contains a carboxyl group and ergotinine is its lactone or lactam. In addition to proving the presence of this carboxyl group in ergotoxine, the authors have been able to demonstrate the presence of a somewhat larger and more characteristic fragment of the complicated molecule of the ergot alkaloids. They have been able to show that the small quantity of a crystalline substance obtained from the destructive distillation of both ergotoxine and ergotinine is *iso*-butyrylformamide.

The Cultivation and Biological Characters of Bacillus Acnes. By H. J. Südmersen, Ph.D., and E. T. Thompson. (From the Wellcome Physiological Research Laboratories, Herne Hill, London, S.E.)

In 1894 Unna and Hodara showed the presence of a bacillus in the comedones of true acne, but were unable to obtain it in pure culture. In 1897 Sabouraud described the organism as being the causative agent of acne and alopecia areata. He cultivated it with some success and drew attention to its extreme variability in form, size, and disposition. In 1909 Fleming described two media on which the organism could be obtained in pure culture, and drew attention to the beneficial effects of treatment by vaccines prepared from the bacillus. The media used were acidulated with HCl and consisted firstly of ordinary agar to which glycerine and oleic acid were added, and secondly of ordinary agar. The present paper is the outcome of the authors' work in the search for a good and simple culture medium and in the investigation of the biological characters of the bacillus. They arrive at the conclusion that a simple medium favourable to the growth of *B. acnes* is easily prepared by the addition of one part of horse-serum to three parts of a 3-per-cent. nutrient agar previously made + 40 acid to phenol-phthalein. The direct cultivation of the organism from the lesion is uniformly successful under anaerobic conditions. After a few generations of anærobic cultivation on acid serum agar growth may be obtained equally well ærobically. The existence of at least two strains of *B. acnes* has been demonstrated. Strain I. has no action on raffinose and very slight action on saccharose, while Strain II. acts strongly on both. Other minor differences in the degree and time of reaction are shown. Remarkable morphological changes are exhibited according to the medium used for its cultivation. A probable relation to *B. diphtheriæ* is shown in many of its characters, and lastly the organism is fatal to mice but not to guinea-pigs.

---

Organic Analysis Chart. By W. Harrison Martindale. (London : H. K. Lewis. 1910. Pp. 80. Price, 3s. 6d. net.)

This is the companion volume to the fourteenth edition of the Extra Pharmacopœia. It is intended to assist in the recognition of a number of organic chemicals, both natural and synthetic, used therapeutically. The data given in the chart are the result of the author's personal laboratory investigations. The little volume should prove useful to analysts and others interested in the investigation of organic compounds.

